# *daf-2* regeneration of mechanosensory neurons: integration

**DOI:** 10.17912/W2NW9C

**Published:** 2017-12-01

**Authors:** Zehra C. Abay, Michelle Yu-Ying Wong, Brent Neumann

**Affiliations:** 1 Neuroscience Program, Monash Biomedicine Discovery Institute and Department of Anatomy and Developmental Biology, Monash University, Melbourne VIC 3800, Australia

**Figure 1. The role of daf-2 in axonal regeneration (Abay et al., 2017b):**
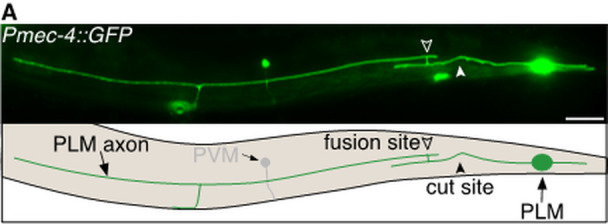
Image and schematic of successful axonal fusion in a posterior lateral microtubule (PLM) neuron 24 h post-axotomy. Closed arrowhead shows cut site, open arrowhead shows fusion site. The posterior ventral microtubule (PVM) neuron is also visible in this image. Scale bar represents 25µm.

## Description

*Caenorhabditis elegans* (*C. elegans*) possess the ability to spontaneously regenerate injured axons via a highly efficient mechanism known as axonal fusion. Following laser axotomy, regrowth from the proximal axon segment (still attached to the cell body) reconnects and fuses with its separated distal segment (Fig. 1). Axonal fusion restores neuronal structure and function (Ghosh-Roy et al., 2010; Neumann et al., 2011; Neumann et al., 2015; Abay et al., 2017). It has been well-established across many different species that advancing age reduces an animals’ capacity for repair after nervous system injury. Although *C. elegans* display a similar decline in terms of the length of axonal regrowth with advancing age, we recently demonstrated that the propensity to repair the mechanosensory neurons via axonal fusion increases with age (Abay et al., 2017a). This increased level of axonal fusion resulted from more robust exposure of injury-induced phosphatidylserine (PS) ‘save-me’ signals that mediate the reconnection, as well as increased regenerative branching, and reduced retraction and degeneration in the separate axon segment.

The *daf-2* gene encodes an insulin-like growth factor/IGF-1 receptor that regulates embryonic and larval development. Mutations in *daf-2* decrease insulin-like signaling, resulting in a 2-fold increase in *C. elegans*lifespan (Kenyon et al., 1993; Kenyon, 2010). It has previously been shown that DAF-2 inhibits neurite regeneration of the GABAergic motor neurons and PVD sensory neurons in an age-dependent fashion (Byrne et al., 2014; Kravstov et al., 2017). To determine if DAF-2 functions in a similar fashion in the mechanosensory neurons to mediate the age-dependent modulation of axonal regrowth and axonal fusion, we studied axonal regeneration in the posterior lateral microtubule (PLM) neurons of animals carrying the *daf-2(e1370)* mutation (Abay et al., 2017b; Abay et al., 2017c).

We quantified the average length of regrowth 24 h post-axotomy in *daf-2* mutants and revealed that *daf-2*modulates PLM regeneration (Abay et al., 2017b). However, instead of delaying the age-dependent reduction in regrowth reported in other neurons (Bryne et al., 2014; Kravtsov et al., 2017), *daf-2* mutants displayed significantly reduced regrowth of PLM at the final larval stage (L4), and in seven-day-old adults (A7). We also showed that mutation of *daf-2* reduced the level of reconnection in A7 animals, but had no effect at other ages (Abay et al., 2017b). As *daf-2* also mediates age-dependent changes in retraction length after transection of the motor neurons (Bryne et al., 2014), we quantified the length of retraction between the severed ends of the PLM axon. This quantification again produced unexpected results, with the length of retraction significantly decreased in L4 stage *daf-2* mutants, but unchanged in adult stages (Abay et al., 2017b). In addition to the increased levels of reconnection that occur with age, we also recently demonstrated that the average number of regenerative branches significantly increases with age (Abay et al., 2017a). The average number of branches was reduced in *daf-2(e1370)* animals across all ages analysed, with significant reductions observed in A5 and A7 animals (Abay et al., 2017b).

We previously established that a critical event for axonal fusion to occur is the exposure of PS ‘save-me’ signals after injury (Neumann et al., 2015), which increase with advancing age (Abay et al., 2017a). To determine if *daf-2* is involved in this age-dependent modulation of PS exposure, we visualised and quantified the level of PS exposed after PLM axotomy using a secreted, tagged version of Annexin V (Neumann et al., 2015; Mapes et al., 2012). We found that there was no effect on PS exposure in *daf-2* mutants 1 h post-axotomy (Abay et al., 2017c).

Overall, our results imply that mutation of *daf-2* does lead to a linear relationship between lifespan extension and modulation of regeneration. This supports the conclusions of (Byrne *et al.* 2014) that insulin signaling regulates axonal regeneration independently from lifespan (Bryne et al., 2014). Our data also further highlights the differences in axonal regeneration that exist between distinct neuronal classes. Although we have recently uncovered many of the molecules and mechanisms required for axonal fusion (Neumann et al., 2015), the genetic mechanisms controlling the age-dependent increase in axonal fusion remain to be defined.
